# Correlating gene and protein expression data using Correlated Factor Analysis

**DOI:** 10.1186/1471-2105-10-272

**Published:** 2009-09-01

**Authors:** Chuen Seng Tan, Agus Salim, Alexander Ploner, Janne Lehtiö, Kee Seng Chia, Yudi Pawitan

**Affiliations:** 1 Lewis-Sigler Institute, Princeton University, New Jersey, USA; 2 Department of Medical Epidemiology and Biostatistics, Karolinska Institutet, Stockholm, Sweden; 3 Center for Molecular Epidemiology, Yong Loo Lin School of Medicine, National University of Singapore and Genome Institute of Singapore, Singapore; 4 Community, Occupational and Family Medicine, Yong Loo Lin School of Medicine, National University of Singapore, Singapore; 5 Cancer Centrum Karolinska, Karolinska Institutet, Stockholm, Sweden; 6 Clinical Proteomics, Karolinska Biomics Center, Karolinska University Hospital, Stockholm, Sweden

## Abstract

**Background:**

Joint analysis of transcriptomic and proteomic data taken from the same samples has the potential to elucidate complex biological mechanisms. Most current methods that integrate these datasets allow for the computation of the correlation between a gene and protein but only after a one-to-one matching of genes and proteins is done. However, genes and proteins are connected via biological pathways and their relationship is not necessarily one-to-one. In this paper, we investigate the use of Correlated Factor Analysis (CFA) for modeling the correlation of genome-scale gene and protein data. Unlike existing approaches, CFA considers all possible gene-protein pairs and utilizes all gene and protein information in its modeling framework. The Generalized Singular Value Decomposition (gSVD) is another method which takes into account all available transcriptomic and proteomic data. Comparison is made between CFA and gSVD.

**Results:**

Our simulation study indicates that the CFA estimates can consistently capture the dominant patterns of correlation between two sets of measurements; in contrast, the gSVD estimates cannot do that. Applied to real cancer data, the list of co-regulated genes and proteins identified by CFA has biologically meaningful interpretation, where both the gene and protein expressions are pointing to the same processes. Among the GO terms for which the genes and proteins are most correlated, we observed blood vessel morphogenesis and development.

**Conclusion:**

We demonstrate that CFA is a useful tool for gene-protein data integration and modeling, where the main question is in finding which patterns of gene expression are most correlated with protein expression.

## Background

### Motivation

Recent advancements in technology have made it possible to jointly analyze a genome-scale gene and protein expression from the same sample. The joint analysis of transcriptomic and proteomic data has potential for shedding new light on complex biological processes. However, the co-analysis of the large datasets continues to present challenges. Some immediate questions are: how does one efficiently characterize the patterns of correlation between the large number of gene and protein expressions? There are many different regulatory pathways within a cell, and many genes and proteins are likely to be co-expressed in a single biological process. How then, can we better identify genes and proteins that are co-regulated, bearing in mind their complex associations? The objective of this paper is to address the above questions.

Although the central dogma of molecular biology suggests a strong correlation between gene and protein expressions, past empirical studies suggest only a modest correlation [1]. Empirical correlations could be masked due to various reasons: the analytical variability of the measurement technologies, post-transcriptional mechanisms affecting mRNA stability and protein degradation, as well as timing differences between gene and protein expressions. Furthermore, it is difficult to find a simple one-to-one relationship for all genes and proteins empirically. Waters *et al*. [2] found that 60% of proteins from liquid chromatography-mass spectrometry (LCMS) analysis do not match the sequence identifiers from two microarray platforms, Affymetrix and Nimblegen. At least 29% and 46% of genes from Affymetrix and Nimblegen, respectively, are not found to match with proteins.

Current methods for the joint analysis of transcriptomic and proteomic datasets entail the matching of genes and proteins through a common identifier from the DNA and protein sequence databases, before computing their pairwise correlations [2,3]. Large amounts of informative data are potentially lost when only genes and proteins with matching sequences are taken into account.

The fact that the proteomic technology is not comprehensive in its coverage also results in loss of informative data: protein expressions corresponding to some genes may not be measured. Various methods have been proposed to deal with the problem. When these unmeasured values are set to zero, Nie *et al*. [4] used the zero-inflated Poisson regression model to account for the excess number of zeros in protein expressions. Imputation, another approach to handle unmeasured or missing value, uses the available information in the proteomic dataset to estimate the missing values. Imputation methods from the microarray literature could be applied to proteomics, for example, weighted K-nearest neighbors [5] and the least-squares principle [6]. To visualize gene and protein expression data, the Co-Inertia Analysis (CIA) was proposed [7]. Nonetheless, these unmeasured protein expressions would result in fewer matched gene-protein pairs, exacerbating the loss of information.

Instead of matching genes and proteins before computing their pairwise correlations, we argue that *all *possible gene-protein pairwise correlations should be considered. This approach is better able to take into account the complex relationships between genes and proteins. For example, genes and proteins that are not matched by the databases could very well be sharing the same pathways. Furthermore, the pooling of gene and protein expressions globally serves to amplify biological signals, thereby improving the chances of discovering the interplay between genes and proteins.

Tractability is an issue when we take into account all possible gene-protein pairwise correlations. We apply the Correlated Factor Analysis (CFA) model, which allows us to characterize succinctly the patterns of global covariation between genes and proteins. In the following subsection, we will show how the CFA framework can be used to model pathways that are shared between genes and proteins. We also applied the CFA to real data from the National Cancer Institute (henceforth NCI data). The next subsection is devoted to describing the analysis done on NCI data. We summarize the contributions of this paper in the final subsection.

### Theoretical correlation model

The cross-covariation matrix of genes and proteins contains information of correlation between all genes and proteins. However, the cross-covariation matrix consists of many parameters. To reduce the number of parameters required to characterize the cross-covariation, we consider a theoretical correlation model. We attempt to capture the complex associations between genes and proteins, taking into account multiple biological pathways. We start with the simplest case of a single pathway.

Let ***x***_*j *_∈ ℝ^*p *^be the column vector of the *p *gene expression values and ***y***_*j *_∈ ℝ^*q *^the column vector of the *q *protein expression values, from sample *j*, for *j *= 1,2,...,*n*.

To reflect a single pathway, we denote the common expression pattern among the genes and proteins as **a **∈ ℝ^*p *^and **b **∈ ℝ^*q *^respectively. If the pathway regulates the first 100 genes and the first 10 proteins, only the first 100 entries of **a **and the first 10 entries of **b **are non-zero. For non-zero entries with high absolute values, their effect on the cross-covariation matrix is greater than those with low absolute values.

Therefore, genes and proteins that are co-expressed and play a greater role in the pathway are likely to have higher absolute values.

From the common expression pattern of the genes and proteins, we could obtain the gene and protein expression values:



where *g*_*i *_∈ ℝ^1 ^and *h*_*j *_∈ ℝ^1 ^are random, and  and  are independent error terms. The cross-covariance between ***x***_*j *_and ***y***_*j *_is given by



so the covariation between ***x***_*j *_and ***y***_*j *_is fully captured by the correlation between unobserved factors *g*_*j *_and *h*_*j*_, and by the pattern-pair **a **and **b**.

With real data we certainly do not expect such a simple representation, but in the same spirit as the principal component analyses, we can expand the model to capture most of the covariation between ***x***_*j *_and ***y***_*j*_. To allow *r *factors, we specify

(1)

where **a**_*k*_s and **b**_*k*_s are gene and protein patterns respectively. Let **A**_*p *× *r *_≡ [**a**_1_...**a**_*r*_] and **B**_*q *× *r *_≡ [**b**_1_...**b**_*r*_], ***g***_*j *_≡ (*g*_*j*1_,...,*g*_*jr*_)' and ***h***_*j *_≡ (*h*_*j1*_,...,*h*_*jr*_)'.

Model (1), called the Correlated Factor Analysis (CFA), is an extension of the standard factor analysis where now the unobserved factors ***g***_*j *_and ***h***_*j *_are designed to be correlated [8]. To avoid non-identifiability we assume orthogonality: **A' A **= *I*_*r *_and **B' B **= *I*_*r*_, and cov(***g***_*j*_, ***h***_*j*_) ≡ **Λ**_*r *× *r *_is diagonal with decreasing positive values [see Additional file 1]. Now the cross-covariance between ***x***_*j *_and ***y***_*j *_is given by

(2)

so the correlation between genes and proteins is characterized by **Λ **and and the pattern-pairs given by **A **and **B **(i.e. *r*(*p *+ *q *+ 1) parameters). Therefore the number of parameters needed to characterize the cross-covariation matrix can be smaller than *pq*.

### Empirical analysis of NCI data

**Figure 1:**
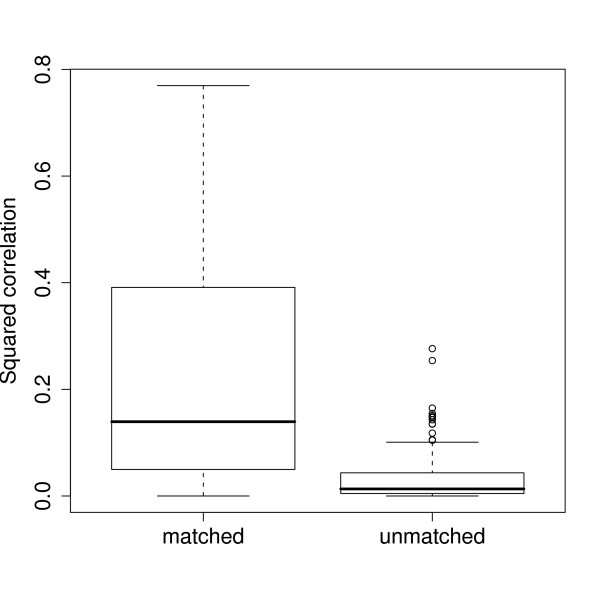
**Boxplots of pairwise *R*^2 ^between gene and protein expressions**. Boxplots of pairwise squared correlations (*R*^2^): 'matched' denotes matched gene-protein pairs, and 'unmatched' denotes unmatched gene-protein pairs.

We observe that there are outliers among the 'unmatched' gene-protein pairs that have correlation values comparable with 'matched' gene-protein pairs; see Figure 1. These outliers could be co-regulated in some common pathway. This supports our proposal to use all genes-proteins pairs instead of limiting analysis to matched gene-protein pairs.

We applied the CFA model (see (1)) to NCI data. The cross-covariance matrix from the CFA model has the same structure as applying the Singular Value Decomposition (SVD) to the cross-covariance matrix. Therefore, it would be possible to obtain the estimates of pattern matrices **A **and **B **quickly via SVD, and avoid using an iterative optimization approach to maximize the likelihood of the CFA model. We used simulated data to verify that SVD provide good estimates for the parameters of the CFA model.

We also compared CFA with another closely related technique called the Generalized Singular Value Decomposition (gSVD). gSVD is used to jointly analyze gene expression and copy number variation information from the same samples [9]. However, the earlier application of the gSVD on two genome-wide datasets were from two independent samples [10]. From simulated and NCI data, we showed that CFA and gSVD produced distinct results.

### Summary contributions

In this paper, we propose a novel approach for jointly analyzing transcriptomic and proteomic data by considering all possible gene-protein pairs. We used a CFA model to succinctly capture the covariation between genome-scale genes and proteins. CFA avoids one-to-one matching of genes and proteins, and utilizes all available information in the analysis. More importantly, CFA considers the global covariation between genes and proteins, and hence pools signals across genes and proteins.

Our proposed approach was applied on real data. We characterized the covariation between genes and proteins using CFA via SVD. We were able to make biological inferences on the selected pattern-pairs by performing an enrichment analysis on the collection of genes.

We found that CFA yielded distinct results from gSVD, an existing technique that is similar to CFA but has been applied on two independent samples.

### Data

#### NCI data

We illustrated the application of CFA and gSVD on real data with the NCI data. Microarray and proteomic datasets from the same human cell line were downloaded from the CellMiner program package developed by National Cancer Institute . Fifty-nine of the 60 human cancer cell lines were used in the analysis as one of the cell lines had missing microarray information. The cell lines consisted of a variety of cancers and were used by the Developmental Therapeutics Program of the U.S. National Cancer Institute to screen more than 100,000 compounds and natural products. For the microarray dataset, we used the Affymetrix HG-U133A chip (Affy) that had been normalized by the GCRMA method [11]. For the proteomic data, reverse-phase protein lysate arrays (RPLA) were used to obtain 89 proteins expression values. These protein values were calculated, with an adjustment for total protein, using the 25% 'dose interpolation' (DI25) algorithm [12]. The dose interpolation for each sample was determined by fitting a monotonic linear spline to the serial dilution curve. The 25% point was chosen because it minimized the measurement variance and yielded more dose-response curves. Each sample's protein DI25 value was normalized by its mean total protein DI25 values.

For the microarray dataset, we excluded genes with variance in the lowest quantile from further analysis, leaving us with 15918 genes. For the proteomic dataset, no filtering was performed. Simple plots of the gene and protein expression values are available [see Additional file 1].

### Simulated data

We performed a simulation study to investigate the consistency of the SVD approach in estimating **a**_**j**_s and **b**_**j**_s in the CFA model, as well as to compare estimates using SVD and gSVD. To simulate correlated gene and protein expression data, we used model (1). In the simulation, we set the sample size to be *n *= 500, with *p *= 1000 genes, *q *= 89 proteins and *r *= 2 pattern-pairs. Results for a small sample of *n *= 59 are also available [see Additional file 1]. To specify realistic gene and protein patterns, and variance parameters, we sub-sampled 1000 genes from the NCI data without replacement and performed a SVD on its cross-covariance matrix. From the SVD of the cross-covariance matrix, we obtained the gene and protein patterns (**A **and **B**).

The factors ()'s are assumed to be iid multivariate normal *N*_2*r*_(**0**, **Ψ**_2*r *× 2*r*_) with



where  and  are assumed to be diagonal matrices. The error terms ()'s are independent of ()' and iid *N*_*p*+*q*_(**0**, **Φ**_(*p*+*q*) × (*p*+*q*)_), where **Φ **is a diagonal matrix. To obtain realistic variance parameters for **Ψ**_*x*_, and **Λ **and **Ψ**_*y*_, we computed the scores of the first two pattern-pairs and their variances. We then computed the residuals and their variances, giving us estimates of **Φ**. Using these estimates, we simulated 250 sets of correlated gene and protein expression datasets.

## Methods

### Notation

Let **X**_*p *× *n *_be the gene expression data matrix from *p *genes measured on *n *samples, and **Y***q *× *n *the proteomic data from *q *proteins from the same *n *samples. We define linear combinations of **X **and **Y **as **u**_**k **_= **X'a**_**k **_and **v**_**k **_= **Y'b**_**k**_. A pattern-pair refers to the vectors **a**_**k **_and **b**_**k**_, which are the gene and protein *patterns *of the *k*-th factor (i.e. *k*-th pattern-pair) of model (1) respectively. The vectors **u**_**k **_and **v**_**k **_are sample gene and protein *scores *associated with the *k*-th pattern-pair. For the NCI data, the dimensions of vectors **a**_**k **_and **b**_**k **_are 15918 and 89 respectively; the dimensions of both vectors **u**_**k **_and **v**_**k **_are 59.

### Obtaining estimates of the pattern-pairs of CFA

To obtain the estimates of pattern matrices **A **and **B **quickly, we performed a Singular Value Decomposition (SVD) on the cross-covariance matrix **Σ**_*p *× *q *_= **XY'**/(*n *- 1), where **X **and **Y **were centered across the rows. If **Σ **is of rank *r*, then by the SVD theorem we have

(3)

where **Λ **and is a diagonal matrix with positive diagonal values arranged in decreasing order. **A **and **B **are orthogonal matrices with the *k*-th column of **A **and **B **corresponding to vector **a**_**k **_and **b**_**k **_respectively. Applying SVD on the cross-covariance matrix provides empirical estimates of the gene and protein patterns in the CFA, i.e. model (1). The relative amount of covariances explained by the *k*-th pattern-pair is .

The sample covariance of the linear combinations of **X **and **Y **with the first pattern-pair, i.e. *λ*_1 _≡ cov(**u**_**1**_, **v**_**1**_) = cov(**X'a**_**1**_, **Y'b**_**1**_), is maximum among all possible choices of **a**_**1 **_and **b**_**1**_, subject to the constraints **a**_**1**_'**a**_**1 **_= 1 and **b**_**1**_'**b**_**1 **_= 1. For the *k*-th pattern-pair, *λ*_*k *_≡ cov(**X'a**_**k**_, **Y'b**_**k**_) is maximized with **a**_**k**_'**a**_**k **_= 1 and **b**_**k**_'**b**_**k **_= 1, subject to **a**_**i**_'**a**_**k **_= 0, ∀*i *<*k*. and **b**_**i**_'**b**_**k **_= 0, ∀*i *<*k*. Therefore, by applying SVD on **Σ**, the sample covariance of the linear combinations of **X **and **Y **is maximized. In the literature, this has been called the Maximum Covariance Analysis (MCA) [13].

How many pattern-pairs should we use? To avoid complex modeling, we used a permutation approach. To obtain the null hypothesis situation of zero cross-covariance (i.e. **Σ **= **0**), we randomly permuted the columns of **Y **(Y*) *P *= 1000 times. SVD was applied to the cross-covariance of **X **and Y*, with singular values  ≥  ≥...≥ . The number of pattern-pairs to use (*k*_0_) was such that the p-value of *λ*_*k *_was less than 0.001 when *k *≤ *k*_0_, and greater than or equal to 0.001 when *k *= *k*_0 _+ 1.

CFA estimates were computed using the functions cov and svd in R statistical programming environment [14], on a PC with 2.66 GHz and 1.95 GB RAM.

### Making biological inferences on the pattern-pairs of CFA

After determining the number of pattern-pairs and obtaining the estimates of the pattern-pairs, the interpretation of these patterns with hundreds or thousands of coefficients (i.e. genes) was non-trivial. Since pattern values with large absolute values had greater influence on the scores, we simplified the interpretation of gene patterns by classifying all 15918 genes into two groups: genes with and without the top *w*% absolute gene pattern values. We classified genes with the top *w*% absolute gene pattern values as interesting genes.

**Figure 2:**
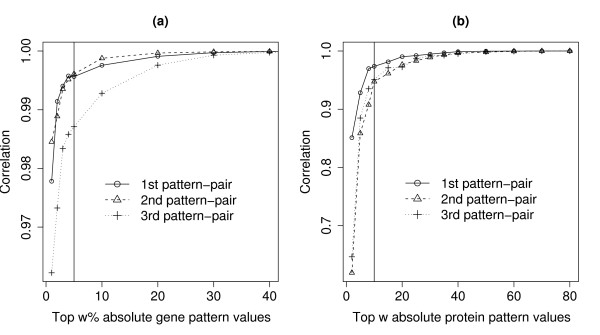
**Plots for determining the optimal value for *w *that classify genes and proteins into interesting or uninteresting sets**. (a) Correlation between the gene scores from SVD based on all pattern values and scores based on the top *w*% absolute gene pattern values at various *w *values. The black vertical line corresponds to 5%. (b) Similar to (a) but for protein scores. The black vertical line corresponds to 10.

The optimal value for *w *was determined by observing its relationship with the correlation between two scores: the first score was computed using the full set of genes and the other using the top *w*% absolute gene pattern values. In Figure 2(a), we plotted the correlation values as a function of *w *for the NCI data. The optimal *w *value corresponded to the 'shoulder' of the curve because genes with negligible contributions should have small absolute coefficients, having minimal impact on the scores computed using the full set of genes. For the NCI data, the optimal value for *w *was five.

The classification of genes into 'interesting' and 'uninteresting' categories facilitated the use of gene ontology (GO) enrichment analysis [15] for biological inferences on each pattern-pair. We performed a GO analysis on the biological processes of genes. The GO analysis tested whether the set of interesting genes was enriched with a particular GO term when compared against all other genes on the microarray. Therefore a GO analysis could be seen as a 2 × 2 table test of association and the Fisher Exact test was used to compute the p-value. To account for multiple testing, GO terms with q-values less than 0.05 were called enriched GO terms [16]. The GO analysis on the molecular functions of genes are available [see Additional file 1].

We also investigated whether gene and protein patterns of the same pattern-pair were extracting biologically coherent signals. The GO terms of proteins from the top *w *absolute protein pattern values were more likely to match the GO terms of gene patterns with the smallest 100 p-values, rather than the bottom *w *absolute protein pattern values. The optimal value for *w *was determined using a similar method used for gene patterns as described earlier. (For protein patterns, *w *was the *number *of proteins and not the percentage of proteins.) For NCI data, the optimal value for *w *was 10; see Figure 2(b). The cut-off for the gene patterns (i.e. smallest 100) was a convenient number that ensured we have sufficient GO terms for matching.

To test whether the gene and protein patterns were giving coherent signals, we ranked the 100 most significant GO terms in descending order of their p-values (i.e. the largest p-value had the lowest rank, while the smallest p-value had the highest rank). For each GO term of a protein, we computed the average rank *M *of p-values from the GO analysis for genes that matched with the protein's GO term. A match between a GO term of a gene and a protein was defined to occur when their GO terms, or the GO terms of their parents, or the GO terms of their children overlap. The Wilcoxon test was used to test whether the median of *M *for the top 10 proteins was significantly different from the bottom 10.

### Comparing CFA with Generalized Singular Value Decomposition (gSVD)

The Generalized Singular Value Decomposition (gSVD) is another method of integrating two datasets. The gSVD simultaneously reduces **X **and **Y **to an *s *× *s *metagene-array space:



where *s *is the rank of [**X', Y'**]'. **A **and **B **are orthogonal matrices. **D**_**X **_and **D**_**Y **_are matrices with (*i*,*j*)-entries having zero values when *i *≠ *j*, and (*i*, *j*)-entries having non-negative values when *i *= *j*. Also, **D**_**X**_'**D**_**X **_+ **D**_**Y**_'**D**_**Y **_= *I*_*s *_[17]. Although **A **and **B **are also the gene and protein patterns for gSVD, they are not the same as their CFA counterparts. The gSVD has a matrix **G **in the expressions of **X **and **Y**, and **G **may be viewed as a link between the two datasets; the columns of **G **are called the generalized singular vectors. Hence **G **potentially captures the correlation between gene and protein expressions. To facilitate a direct comparison of the matrices **A **and **B **between CFA and gSVD, we did not use the iterative gSVD by Berger *et al*. [9] in this paper.

The *i*-th row of **G**^-1 ^contains the expression values of the *i*-th metagene across the *n *arrays, while the *j*-th column of **A **and **B **are the expression values of the *j*-th meta-array across the genes and proteins respectively. Each of the metagene-array pair may represent independent biological processes. Similar to CFA, the significance of the *i*-th metagene and its corresponding meta-array for dataset *j *= **X, Y **is quantified by

(4)

where *d*_**X***i *_and *d*_**Y***i *_are the (*i*, *i*)-entries of **D**_**X **_and **D**_**Y **_respectively, and they carry the expression information of the *i*-th metagene and its corresponding meta-array in **X **and **Y**. *P*_*ij *_is also called the generalized variance explained for the *j*-th dataset [9].

The relative significance of the *i*-th metagene was assessed through the ratio of the expression information from the datasets [10]:



When the angular distance, *θ*_*i*_, is 0, the *i*-th metagene may be equally significant in both datasets.

However, when the angular distance is *π*/4, the *i*-th metagene may have no significance in **Y **relative to **X**. And when the angular distance is -*π*/4, the *i*-th metagene may have no significance in **X **relative to **Y**. Hence metagenes with the smallest angles best captured the gene-protein correlation. To identify the genes that may have parallel contribution for **X **and **Y **in a metagene, we used the metagene's corresponding meta-array expression values (i.e. pattern-pairs), and declared genes with the top 5% absolute gene pattern values as the main contributors to the metagene.

We also made biological inferences on gSVD pattern-pairs using the same approach described in the previous subsection 'Making biological inferences on the pattern-pairs of CFA'. To compare how well CFA and gSVD extracted coherent signals from the genes and proteins, we constructed a measure for determining the similarity between the GO terms from genes and proteins. Using the top and bottom 10 absolute protein pattern values respectively, we obtained two lists of GO terms. Similarly, we generated two lists of GO terms from genes which had the same number of GO terms as the proteins and had the lowest p-values from the GO analysis. The GO terms of each list were building blocks for each induced GO graph. Besides the list of GO terms, each induced graph also consisted of all the ancestors of the GO terms back to the root node. Although each induced graph was built upon the same number of GO terms, the number of nodes from the protein's induced GO graph was on average three times more than the genes. One possible explanation was that the protein data are far less comprehensive than the gene data, so there was less overlap among the protein nodes. Therefore, we used the proportion of nodes from the gene's induced graph that overlapped the nodes from the protein's induced graph as a measure of similarity between the GO terms from genes and proteins.

The gSVD estimates were computed using the Lapack fortran package in R, but for the NCI data, we ran the gSVD analysis on a supercomputer.

## Results

### Applying CFA to simulated data

**Figure 3:**
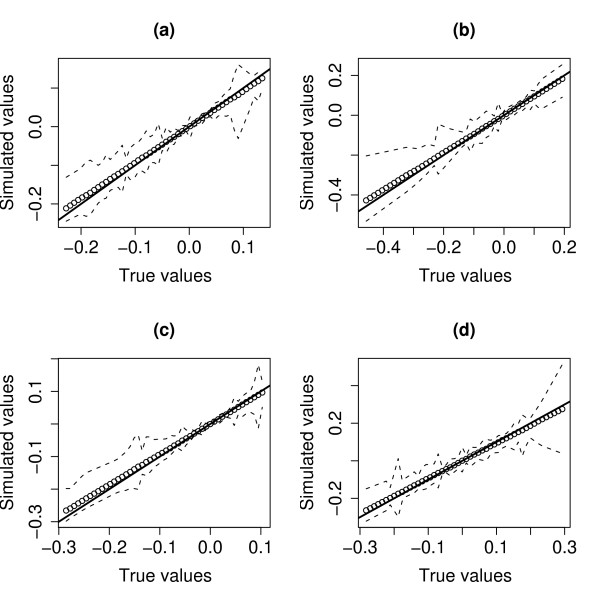
**Plots of true pattern-pairs versus estimated pattern-pairs from CFA by using SVD**. True pattern-pairs versus estimated pattern-pairs from CFA by using SVD (250 simulations with *n *= 500 samples). (a) and (b) are the gene and protein patterns of the first pattern-pair respectively, while (c) and (d) are the gene and protein patterns of the second pattern-pair respectively. The solid line is the line-of-identity, the broken line is the interpolated 5th and 95th percentile of the estimated patterns from 250 simulations, while the circles are their interpolated means.

Figure 3 shows the true pattern-pairs versus the pattern-pairs from CFA obtained by SVD for *n *= 500, summarized from 250 replications. (The signs of the pattern-pairs from CFA were reversed when their correlation coefficients with the true pattern-pairs were negative for both genes and proteins.) Figure 3(a) and 3(b) are the gene and protein patterns of the first pattern-pair respectively; Figure 3(c) and 3(d) are the gene and protein patterns of the second pattern-pair respectively. The results suggest that SVD produced consistent estimates of the gene and protein patterns. In small samples (*n *= 59), however, we observed a slight bias [see Additional file 1].

### Applying gSVD to simulated data

**Figure 4:**
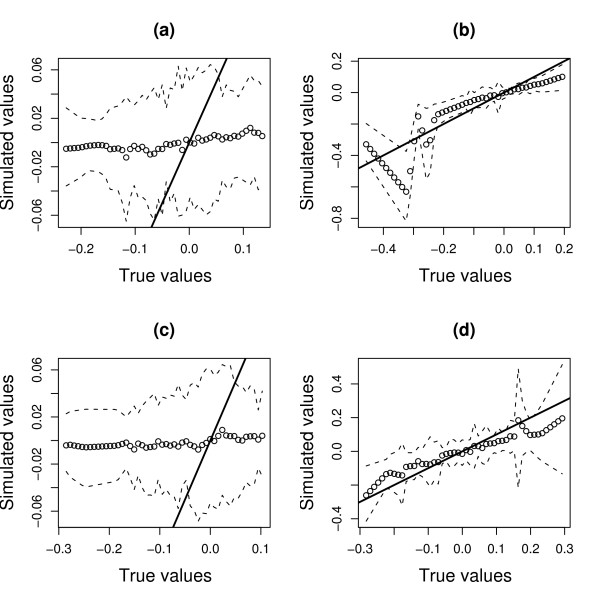
**Plots of true pattern-pairs versus estimated pattern-pairs from gSVD having the highest absolute correlation with the true patterns**. True pattern-pairs versus estimated patterns from gSVD having the highest absolute correlation with the true patterns (250 simulations with *n *= 500 samples). (a) and (b) are the gene and protein patterns of the first pattern-pair respectively, while (c) and (d) are the gene and protein patterns of the second pattern-pair respectively. The solid line is the line-of-identity, the broken line is the interpolated 5th and 95th percentile of the estimated patterns from 250 simulations, while the circles are their interpolated means.

We investigated whether gSVD can estimate the true patterns by considering gene and protein patterns which had the highest absolute correlation with the corresponding true patterns. The results for *n *= 500 given in Figure 4 indicates that gSVD captured some correlation patterns in the data, particularly the protein patterns. However, the estimates were not consistent. Furthermore, among the gene and protein patterns with the highest absolute correlation, only 11% of them were of the same pattern-pair.

We investigated if the angular distance improved the strength of the correlation between the pattern estimates from gSVD and the true patterns. There was no evidence of improvement [see Additional file 1].

### Applying CFA to NCI data

**Figure 5:**
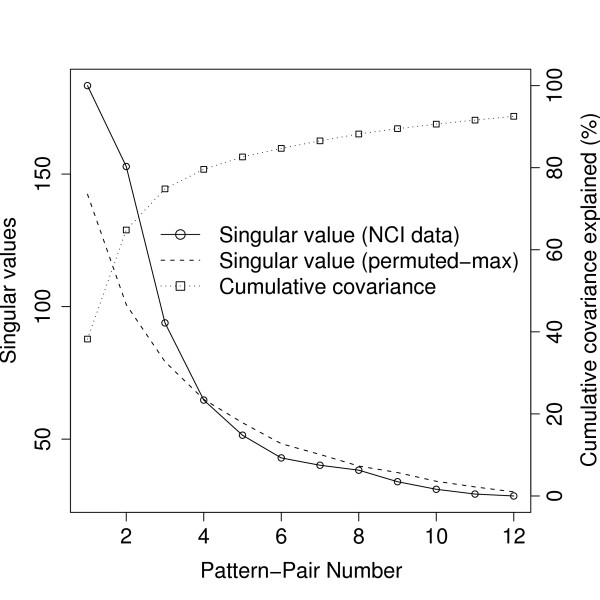
**Plots to determine the number of pattern-pairs of the NCI data analysis using CFA**. The observed singular values from the NCI data (solid line with circles), maximum singular values from 1000 permutations (broken line) and cumulative covariance (dotted line with squares) profiles of the 1-th to 12-th pattern-pairs.

We first determined the number of pattern-pairs through the permutation approach. Figure 5 shows the singular value and cumulative variance profile for the 1st to 12th pattern-pairs. The first three consecutive singular values from the data (solid lines with circles) were larger than the maximum singular value from the permutations (dashed lines), i.e. these singular values have p-values < 0.001. The *R*^2 ^between the gene and protein patterns within each pattern-pair were 0.81, 0.88, 0.75, respectively, substantially larger than the median *R*^2 ^in Figure 1. From the cumulative profile (dotted lines with squares), we observed that the first three pattern-pairs explained 74.8% of the covariation, and the curve started to plateau off to 100% for subsequent pattern-pairs. Hence, the first three pattern-pairs were adequate in capturing the structure of the cross-covariance matrix between genes and proteins.

After determining the number of pattern-pairs, we selected the genes that were interesting within each pattern-pair, by identifying the top 5% of absolute gene pattern values within each pair. Among the chosen genes from the first three pattern-pairs (1655 genes), about a third of them (566 genes) were also selected in another pattern-pair. This was reasonable as proteins and genes could be involved in a few different pathways.

To gain biological insights into the sets of interesting genes from each pattern-pair, we performed a GO analysis on the biological processes. The q-value cut-off was set at 0.05 for evaluating over-representation of biological processes (i.e. enriched GO terms). The number of GO terms and the corresponding number of enriched GO terms for the genes were 1827 and 106 for the first gene patterns, 1681 and 127 for the second gene patterns, and 1999 and 141 for the third gene patterns. There were altogether 240 enriched GO terms and about a third of them (89 GO terms) were also interesting in another pattern-pair.

Table 1 shows the top 10 most enriched GO terms from the genes for each pattern-pair. The unique GO terms of the first pattern-pair from CFA were blood vessel morphogenesis and development, which are processes associated in the growth of primary solid tumors and the invasive property of the tumor [18]. The unique GO terms of the second pattern-pair from CFA were cell motility and localization of cell, which are processes related to the malignant potential of a tumor in prostate cancer [19]. The unique GO terms of the third pattern-pair from CFA were mainly melanin biosynthetic processes, which are associated with the risk of skin cancer [20].

**Table 1:** Enriched biological process GO terms from genes.

GO ID	GO Term	Q-value(1)	Q-value(2)	Q-value(3)
GO:0048514	blood vessel morphogenesis	3*:*50*e *- 09		
GO:0001568	blood vessel development	6*:*19*e *- 08		
GO:0006928	cell motility		3*:*57*e *- 10	
GO:0051674	localization of cell		3*:*57*e *- 10	
GO:0009653	anatomical structure morphogenesis			4*:*08*e *- 06
GO:0006582	melanin metabolic process			6*:*61*e *- 04
GO:0006583	melanin biosynthetic process from tyrosine			6*:*61*e *- 04
GO:0042438	melanin biosynthetic process			6*:*61*e *- 04
GO:0030154	cell differentiation			9*:*97*e *- 04
GO:0048731	system development	9*:*62*e *- 12	4*:*87*e *- 14	
GO:0007275	multicellular organismal development	1*:*03*e *- 11	1*:*00*e *- 12	
GO:0048513	organ development	9*:*60*e *- 10	8*:*81*e *- 11	
GO:0048856	anatomical structure development	3*:*12*e *- 13	1*:*14*e *- 17	1*:*79*e *- 05
GO:0032501	multicellular organismal process	3*:*12*e *- 13	4*:*99*e *- 14	6*:*39*e *- 05
GO:0032502	developmental process	6*:*42*e *- 13	9*:*34*e *- 16	6*:*34*e *- 04
GO:0007155	cell adhesion	3*:*21*e *- 10	1*:*00*e *- 12	9*:*31*e *- 06
GO:0022610	biological adhesion	3*:*21*e *- 10	1*:*00*e *- 12	9*:*31*e *- 06

The top 10 most enriched biological process GO terms from genes for the first three pattern-pairs from CFA.

The top corresponding proteins are given in Table 2. A number of proteins were unique for each pattern, and CFA suggested that there was a strong association between these proteins and the corresponding processes in Table 1. Focusing on the first pattern-pair, its enriched GO terms, blood vessel morphogenesis and development, had descendants with annotations that overlap with NP_001895.1, which is an adherens junction protein. This protein regulates normal cell growth and behavior, but when down-regulated it causes increased invasiveness and metastatic potential of tumors, which is associated with blood vessel morphogenesis and development. This was consistent with the NCI data, which consisted of cancer cell lines. This suggests that CFA has the potential to extract biologically meaningful pairs of genes and proteins in the same pathway.

**Table 2:** The top 10 proteins from the NCI data analysis using CFA.

Ref Seq	Name	Rank(1)	Rank(2)	Rank(3)
NP_005547.3	keratin 7	4		
NP_003370.2	ezrin	9		
NP_061883.1	keratin 20	10		
NP_057011.2	transforming growth factor beta 1 induced transcript 1 isoform 2		1	
NP_001783.2	cadherin 2, type 1 preproprotein		3	
NP_002378.1	mutated in colorectal cancers isoform 2		6	
NP_001144.1	annexin IV		7	
NP_478104.2	cyclin-dependent kinase inhibitor 2A isoform 3		8	
NP_005222.2	cortactin isoform a		9	
NP_002435.1	moesin			2
NP_002728.1	protein kinase C, alpha			4
NP_000606.3	neural cell adhesion molecule 1 isoform 1			5
NP_004351.1	cadherin 1, type 1 preproprotein			6
NP_000691.1	annexin I			8
NP_954657.1	keratin 18	1	2	
NP_001002858.1	annexin A2 isoform	3	4	
NP_002267.2	keratin 19	2		1
NP_001895.1	catenin (cadherin-associated protein), beta 1, 88 kDa	6		7
NP_002764.1	prostasin preproprotein	7		10
NP_002264.1	keratin 8	5	10	3
NP_997700.1	protein kinase C, beta isoform 1	8	5	9

The 10 proteins with the largest absolute protein pattern values from the NCI data analysis using CFA.

Next, we tested whether the pattern-pairs from CFA produce coherent signals. If they did, it would indicate that CFA was able to extract true biological signals. Each GO term of a protein from the top or bottom 10 absolute protein patterns was matched with the GO terms from the genes with the lowest 100 p-values.

**Figure 6:**
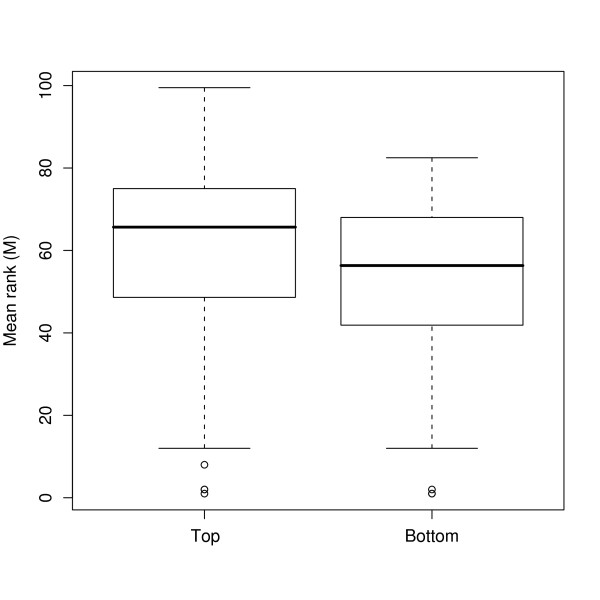
**Boxplot of the average rank of p-values from the GO analysis of the NCI data analysis using CFA**. The boxplot of the average rank *M *of p-values, which are from the GO analysis on the biological process of genes, for protein GO terms that are from the top 10 and bottom 10 absolute protein patterns.

For each protein's GO term, we computed the average rank *M *of p-values from the GO enrichment analysis for genes that matched with the protein's GO term. Figure 6 shows that the protein GO terms associated with the top 10 proteins were more highly significant than those from the bottom 10 proteins (p = 0.009 using the Wilcoxon test), suggesting a concordance between the gene and protein patterns. In other words, the gene and protein pattern-pairs from CFA were extracting similar biological signals.

### Applying gSVD to NCI data

**Figure 7:**
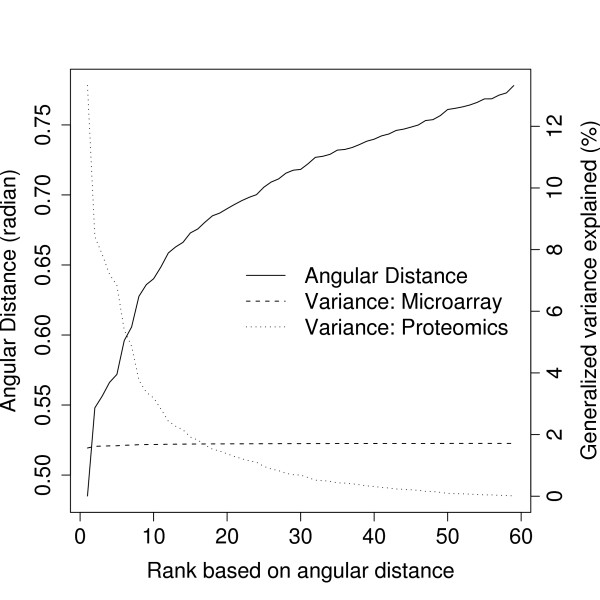
**Ranked angular distance and generalized variance of the NCI data analysis using gSVD**. The ranked angular distance profile (solid line) together with their generalized variance explained by the metagene-array pairs for the microarray (dashed line) and proteomic data (dotted line).

We attempted to determine the interesting metagene-array or pattern-pairs using the angular distance. From Figure 7, we obtained the profile of the angular distance (solid line), the generalized-variance explained corresponding to the microarray (dashed line) and proteomic data (dotted line). All 59 angular distances were positive and ranged from 0.485 to 0.778. The generalized variance explained for the microarray data was quite uniform across metagene-array pairs, while the generalized variance explained for the proteomic data was high when the angular distance was low. In view of the generalized variance explained, we further investigated the pattern-pairs with the lowest three angular distances (0.485, 0.548 and 0.556).

Similar to the CFA, we chose genes from the top 5% absolute gene pattern values. Among the 1952 selected genes from the three pattern-pairs with low angular distances, about 20% of them (393 genes) were also chosen in another pattern-pair. Similar to the CFA, we performed a GO analysis [see Additional file 1]. One of the unique GO terms of the first pattern-pair was transmembrane receptor protein tyrosine kinase signaling pathway, which is upstream of the PI3K pathway; a pathway associated with cancer [21]. Some of the unique GO terms of the first pattern-pair were similar to the unique GO terms of the first pattern-pair of CFA. The second pattern-pair did not contain GO terms that are unique to itself. The unique GO terms of the third pattern-pair were response to external stimulus and descendants of anatomical structure development.

We analyzed the concordance between the gene and protein patterns as detailed in the previous subsection. The median of the average p-value rankings of the GO terms (*M*) from the top 10 proteins was lower than the bottom 10 but insignificant (p = 0.130 using the Wilcoxon test). Although there is insufficient evidence that gSVD gene and protein pairs were internally incoherent, the genes and proteins could be referring to different processes.

### Comparing CFA and gSVD with NCI data

We tried to match CFA and gSVD results as much as possible by identifying pattern-pairs from gSVD that had the highest absolute correlation with the first three pattern-pairs from CFA. Interestingly, for the first pattern-pair from CFA, the identified gene and protein patterns from gSVD were not from the same pair: they were from the 7th and 3rd pairs respectively. However, the gene and protein patterns from gSVD having the second highest absolute correlation were from the 3rd and 7th pairs respectively. This suggests a mis-pairing. Only the 3rd pattern-pair from gSVD was investigated here, as the 7th pattern-pair had the smallest angular distance. For the second and third pattern-pairs from CFA, the identified gene and protein patterns from gSVD with the highest absolute correlation were from the same pairs.

Similar to the previous subsections, we chose genes from the top 5% absolute gene pattern values. Among the 1877 genes from the three pattern-pairs, about a quarter of them (454 genes) were also interesting in another pattern-pair. Similar to the previous subsections, we performed a GO analysis [see Additional file 1]. We also analyzed the concordance between the gene and protein patterns. The median of the average p-value rankings of the GO terms (*M*) from the top 10 proteins was significantly different from the bottom 10 proteins (p = 0.019). This indicates that these gSVD gene and protein pairs were potentially internally coherent when correlated with CFA.

**Figure 8:**
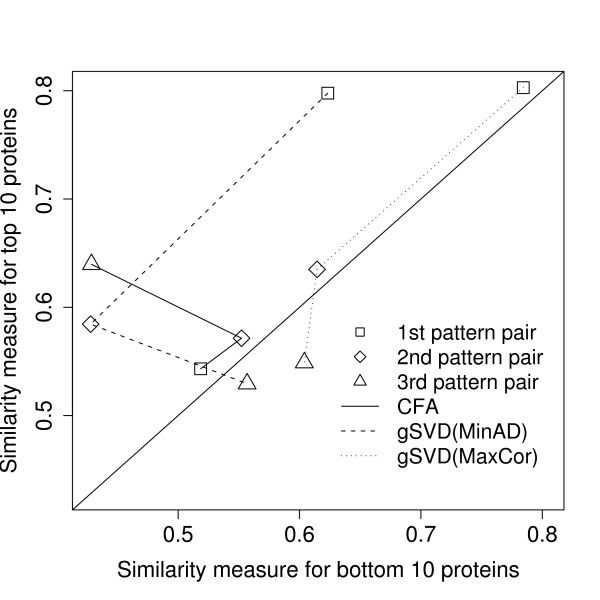
**Comparing the similarity measure of CFA and gSVD**. The proportion of nodes from the gene's induced graph overlapping with the nodes from the protein's induced graph (similarity measure) for the top and bottom 10 proteins of CFA and gSVD. The diagonal line is where the values from the x-axis and y-axis are equal. gSVD(MinAD): gSVD with the smallest angular distances, gSVD(MaxCor): gSVD having the highest correlation with the first three pattern-pairs from CFA.

Figure 8 plots the similarity measure of the GO terms from genes, and GO terms from proteins with the top and bottom 10 absolute protein patterns. The points are similarity measures of the different pattern-pairs (square = first, diamond = second, triangle = third) and different approaches (solid line = CFA, dashed line = gSVD with the smallest angular distances, dotted line = gSVD having the highest correlation with the first three pattern-pairs from CFA). Because the top 10 absolute protein patterns should match the more highly significant GO terms from genes than the bottom 10, most of the points should ideally be above the line of identity. Only all the points from CFA (connected by the bold line) were above the diagonal line, indicating consistent concordance between the gene and protein GO terms in all three pattern-pairs. However, the gSVD approaches had pattern-pairs with a higher similarity measure than CFA.

## Discussion

The CFA model was used to capture the complex associations between genes and proteins, taking into account multiple biological pathways. We were able to obtain consistent estimates of the pattern-pairs of CFA, as indicated in the results of our simulation study. NCI data were made up of cancer cell lines and our results showed that the unique GO terms of each pattern-pair were indeed cancer related.

Furthermore, there was biological coherence in the pattern-pairs, i.e. the genes and proteins in a pair were pointing to the same biological processes (as defined by the Gene Ontology).

On the other hand, our simulation study indicated that gSVD did not capture the specified pattern-pairs. Although the results from applying gSVD to NCI data suggested that gSVD was able to capture biological signals, there seemed to be no biological coherence in the pattern-pairs of gSVD.

Some of the GO terms from gSVD were different from CFA, indicating differences between pattern-pairs of CFA and gSVD. This is because gSVD is a generalization of principal component analysis (e.g. maximizing the variability of linear combinations of expression values, or looking for the direction with maximum variability), while in contrast, CFA looks for linear combinations that have maximal covariance. gSVD is connected to the generalized eigenvalue problem and therefore, if *Y *is of rank *n*, which is usually the case in most applications, then the eigenvectors of *X'X *- *λY'Y *are the same as the eigenvectors of *X'X*(*Y'Y*)^-1^. This indicates that gSVD is not designed to search for correlation between two sets of measurements. From gSVD, we could get the following equation:

(5)

From (5), we see that gSVD does not decompose the cross-covariance matrix of genes and proteins. This explains why the pattern-pairs were different for the two methods. gSVD is decomposing an expression similar to a cross-covariance matrix estimate of the modified gene and protein expressions matrices (i.e. **XG **and **YG**). Therefore, gSVD could capture some portion of the correlation between genes and proteins. However, if capturing the correlation between gene and protein expressions is the main purpose, then CFA may be more effective as it is designed to do so.

Both the simulation study and real data analyses showed that CFA revealed the underlying correlation between gene and protein expressions, while gSVD did not. Nonetheless, relating CFA and gSVD by extending gSVD to model the cross-covariation is a research area worth exploring.

The SVD is commonly used to characterize variation in a single phenotype, such as gene expression [22]. In this paper, we extend the use of SVD to characterize correlation between two phenotypes (i.e. gene and protein expressions). Interestingly, the cross-covariance matrix from the CFA model has the same structure as applying the SVD to the cross-covariance matrix. Therefore, we can adapt CFA immediately to empirical data via SVD, and this provides another characterization of the SVD analysis. To understand the correlation between genes and proteins in terms of some underlying factors, we need measurements from the *same samples*. If the SVD technique is applied to genes and proteins from different samples, it is addressing questions other than correlation.

Analysis of more than two datasets is an interesting extension. In principle we can expand CFA model in the same spirit as the multiple factor analysis [23], but the computation using SVD is no longer obvious.

## Conclusion

For correlating transcriptomic and proteomic data, we found that CFA was more appealing than the current integrative approach. This is because it allowed proteins to correlate throughout the genome, reflecting the biological phenomenon of genes being connected in various pathways. Furthermore, CFA circumvented the step to match genes and proteins, and exploited all information in the analysis, so increasing the chances of uncovering biologically novel relationship between genes and proteins. We compared CFA and gSVD using simulated and real data, and showed that CFA captured the underlying correlation between gene and protein expressions, while gSVD did not.

## Authors' contributions

All authors contributed to the approach of the analysis. CST performed the analysis and drafted the manuscript. AS, AP, JF and KSC revised the manuscript. YP supervised the analysis, provided oversight and revised the manuscript. All authors have read and approved the manuscript.

## Supplementary Material

Additional file 1 Additional material to the paper Click here for file
