# Supplementary material for: Correlating gene and protein expression data using Correlated Factor Analysis
Source: BMC Bioinformatics. 2009 Sep 1;10:272. doi: 10.1186/1471-2105-10-272 (PMC2744708; doi:10.1186/1471-2105-10-272)
Supplement: Additional file 1 — Additional material to the paper [file 1471-2105-10-272-S1.pdf]

Additional file 1: Correlating gene and protein expression data using Correlated Factor Analysis

By Chuen Seng Tan, *et al.*

1 Proof of identifiability for Correlated Factor Analysis (CFA)

This section gives the proof that the Correlated Factor Analysis (CFA) model is unique when we assume orthogonality: $\mathbf{A}'\mathbf{A} = I_r$ and $\mathbf{B}'\mathbf{B} = I_r$, and $\text{cov}(\boldsymbol{g}, \boldsymbol{h}) \equiv \boldsymbol{\Lambda}_{r\times r}$ is diagonal with decreasing values.

Theorem 1 *Given the r-factor Correlated Factor Analysis (CFA) model*

$$\boldsymbol{z}_{j_{(p+q)\times 1}} = \mathbf{L}_{(p+q)\times 2r}\mathbf{f}_{j_{2r\times 1}} + \boldsymbol{\epsilon}_{j_{(p+q)\times 1}}$$

where $\boldsymbol{z}_j \equiv (\boldsymbol{x}_j', \boldsymbol{y}_j')'$, $\mathbf{f}_j \sim N_{2r}(0, \boldsymbol{\Psi}_{2r\times 2r})$, $\boldsymbol{\epsilon}_j \sim N_{(p+q)}(0, \boldsymbol{\Phi}_{(p+q)\times(p+q)})$ *and* $\mathbf{L}'\mathbf{L} = I_{2r}$ *and* $\boldsymbol{\Psi}$ *can be partitioned as*

$$\boldsymbol{\Psi}_{2r\times 2r} = \left(\begin{array}{c|c} \boldsymbol{\Psi}_{x_{r\times r}} & \boldsymbol{\Lambda}_{r\times r} \\ \hline \boldsymbol{\Lambda}_{r\times r} & \boldsymbol{\Psi}_{y_{r\times r}} \end{array}\right),$$

where $\boldsymbol{\Lambda}$ *is diagonal and its main diagonal elements are ordered such that* $\boldsymbol{\Lambda} = diag\{\lambda_1, \lambda_2, \ldots, \lambda_r\}$ *with* $\lambda_1 > \lambda_2, \ldots, > \lambda_r > 0$.

If the loading matrix ($\mathbf{L}$*), factor variables (*$\mathbf{f}_j s$*) and errors variables (*$\boldsymbol{\epsilon}_j s$*) are given by:*

$$\mathbf{L} = \left(\begin{array}{c|c} \mathbf{A} & 0 \\ \hline 0 & \mathbf{B} \end{array}\right),$$

$$\mathbf{f}_j = \left(\begin{array}{c} \boldsymbol{g}_j \\ \hline \boldsymbol{h}_j \end{array}\right), \text{ and}$$

$$\boldsymbol{\epsilon}_j = \left(\begin{array}{c} \boldsymbol{\epsilon}_j^x \\ \hline \boldsymbol{\epsilon}_j^y \end{array}\right),$$

then the parameters are unique.

Proof: Suppose the estimates are not unique. In other words, there exists a non-singular matrix $\mathbf{C}$ so that a new loading matrix $\mathbf{L}^* = \mathbf{LC}$ and new factor covariance matrix $\boldsymbol{\Psi}^* = \mathbf{C}^{-1}\boldsymbol{\Psi}\mathbf{C}'^{-1}$ satisfy the constraints of the model, $\mathbf{L}^{*\prime}\mathbf{L}^* = I_{2r}$.

Suppose we partitioned matrix C into

$$\mathbf{C}_{2r\times 2r} = \left(\begin{array}{c|c} \mathbf{C}_{11_{r\times r}} & \mathbf{C}_{12_{r\times r}} \\ \hline \mathbf{C}_{21_{r\times r}} & \mathbf{C}_{22_{r\times r}} \end{array}\right).$$

The new loading matrix $\mathbf{L}^*$ is given by

$$\mathbf{L}^* = \left(\begin{array}{c|c} \mathbf{AC}_{11} & \mathbf{AC}_{12} \\ \hline \mathbf{BC}_{21} & \mathbf{BC}_{22} \end{array}\right).$$

In order for $\mathbf{L}^*$ to have the same structure as $\mathbf{L}$ then $\mathbf{AC}_{12}$ and $\mathbf{BC}_{21}$ must be zero. But since columns of $\mathbf{A}$ and $\mathbf{B}$ are mutually orthogonal this is only possible if $\mathbf{C}_{12} = \mathbf{C}_{21} = 0$. The orthogonality constraints also have to be preserved, this means $\mathbf{C}'_{11}\mathbf{A}'\mathbf{A}\mathbf{C}_{11} = \mathbf{C}'_{22}\mathbf{B}'\mathbf{B}\mathbf{C}_{22} = I_r$. But since $\mathbf{A}'\mathbf{A} = \mathbf{B}'\mathbf{B} = I_r$, matrices $\mathbf{C}_{11}$ and $\mathbf{C}_{22}$ which satisfy the orthogonality constraint must be orthogonal.

The new factor covariance matrix $\mathbf{\Psi}^*$ can be written as

$$\mathbf{C}^{-1}\mathbf{\Psi}\mathbf{C}'^{-1} = \left(\begin{array}{c|c} \mathbf{C}_{11}^{-1}\mathbf{\Psi}_x\mathbf{C}_{11}'^{-1} & \mathbf{C}_{11}^{-1}\mathbf{\Lambda}\mathbf{C}_{22}'^{-1} \\ \hline \mathbf{C}_{22}^{-1}\mathbf{\Lambda}\mathbf{C}_{11}'^{-1} & \mathbf{C}_{22}^{-1}\mathbf{\Psi}_y\mathbf{C}_{22}'^{-1} \end{array}\right).$$

$\mathbf{C}_{11}^{-1}\mathbf{\Lambda}\mathbf{C}_{22}'^{-1}$ and $\mathbf{C}_{22}^{-1}\mathbf{\Lambda}\mathbf{C}_{11}'^{-1}$ are diagonal matrices, denoted as $\mathbf{\Lambda}^*$. Since $\mathbf{\Lambda} = \mathbf{C}_{11}\mathbf{\Lambda}^*\mathbf{C}'_{22}$, $\mathbf{C}_{11}\mathbf{\Lambda}^*\mathbf{C}'_{22}$ is the SVD representation of $\mathbf{\Lambda}$, where $\mathbf{\Lambda} = \text{diag}\{\lambda_1, \lambda_2, \ldots, \lambda_r\}$ with $\lambda_1 > \lambda_2, \ldots, > \lambda_r > 0$. Therefore $\mathbf{C}_{11}$ and $\mathbf{C}_{22}$ are identity matrices, and $\mathbf{\Lambda}^* = \mathbf{\Lambda}$. Hence the solution is unique.

2 NCI microarray data

59 of the 60 human cancer cell lines Affymetrix HG-U133A chip from National Cancer Institute (NCI) were used in this paper. The gene expression values used here had been normalized by the GCRMA method. We further filtered out genes with low variation in this analysis. Figure 1 shows the qq-plot of the gene expression values of the 59 samples after filtering, where Figure 1 (a) is for all samples, while (b)-(d) are for three samples respectively. The boxplot of the gene expression values for each sample is in Figure 2.

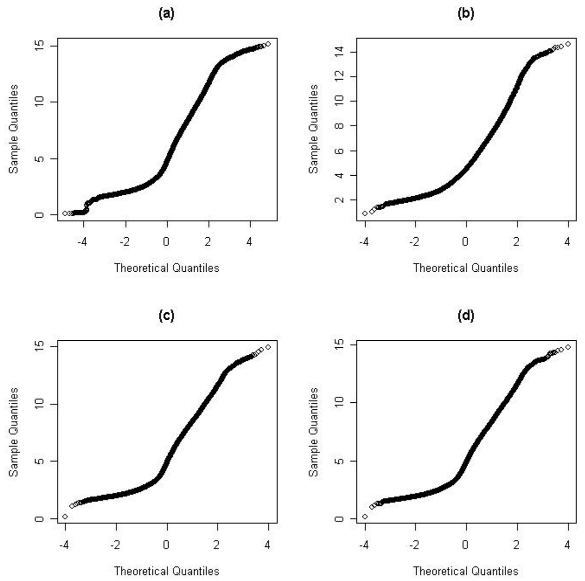

Figure 1: The qq-plot of the gene expressions from: (a) all 59 samples, and (b)-(d) three samples respectively.

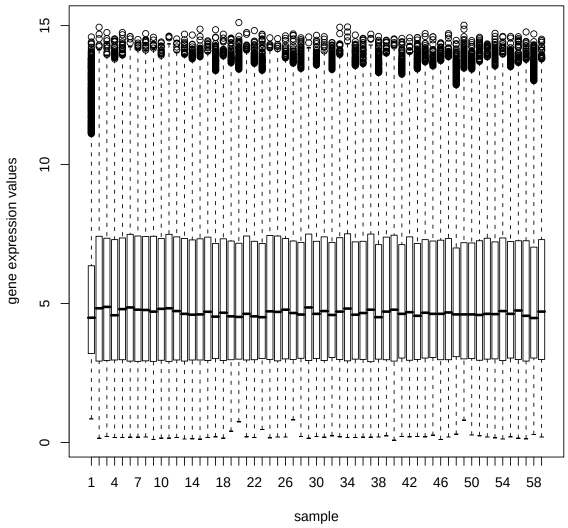

Figure 2: The boxplots of the gene expressions from the 59 samples.

3 NCI protein data

59 of the 60 human cancer cell lines reverse-phase protein lysate arrays (RPLA) from National Cancer Institute (NCI) were used in this paper. The protein expression values used here had been condensed into 89 proteins expression values and normalized. For the proteomic dataset, no filtering was performed. The qq-plot of the protein expression values from the 59 samples are in Figure 3, where (a) is for all samples, while (b)-(d) are for three samples respectively, and the boxplot of the protein expression values for each sample are in Figure 4.

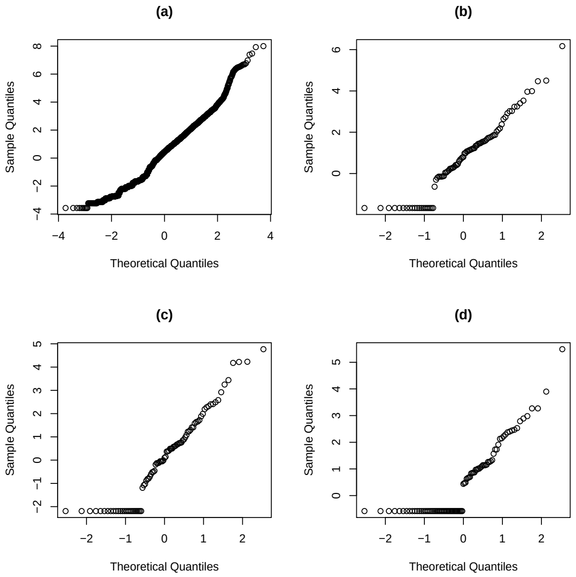

Figure 3: The qq-plot of the protein expressions from: (a) all 59 samples, and (b)-(d) three samples respectively.

4 Simulated data results of CFA

In this section, the simulation was based on 59 samples. Figure 5 (a) and (b) are the gene and protein patterns of the first pattern-pair respectively, while Figure 5 (c) and (d) are the gene and protein patterns of the second pattern-pair respectively. The solid line is the line-of-identity, the broken line is the interpolated 5-th and 95-th percentile of the patterns from 250 simulations, while the circles are their interpolated mean patterns. The patterns from CFA via SVD were slightly away from the line-of-identity, indicating a slight bias.

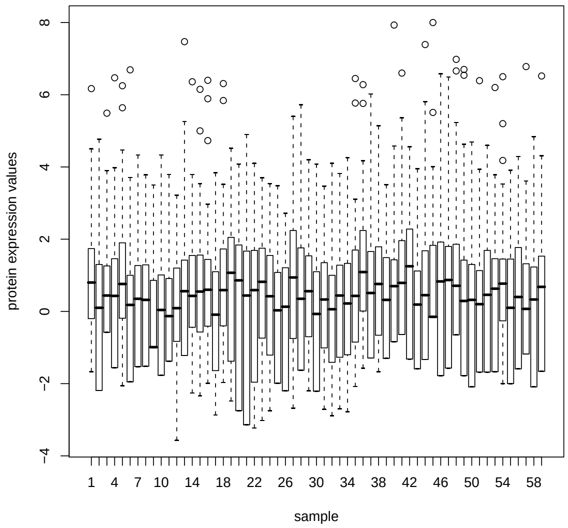

Figure 4: The boxplots of the protein expressions from the 59 samples.

5 Simulated data results of gSVD

To investigate whether the estimated patterns from gSVD can identify the true patterns in the simulation with 59 samples, we plotted the estimated patterns from gSVD having the highest absolute correlation with the true patterns; see Figure 6. Figure 6 (a) and (b) are the gene and protein patterns of the first pattern-pair respectively, while Figure 6 (c) and (d) are the gene and protein patterns of the second pattern-pair respectively. The patterns from gSVD were biased.

We also investigated if the angular distance improved the strength of the correlation between the estimated patterns from gSVD and the true patterns when $n = 59$, because the bias was less than $n = 500$. Since the range of the angular distances was -0.129 and 0.746, we considered angular distances near $\pi/4$ and 0. Among the two pattern-pairs with angular distance nearest to $\pi/4$ and 0 respectively, the one having the highest absolute correlation with the true patterns was used. Figure 7 are the boxplots of the: (i) estimated patterns from gSVD having the largest absolute correlation with the true patterns, Cor (max), (ii) estimated patterns from gSVD having the highest absolute correlation with the true patterns among the two pattern-pairs with angular distances nearest to $\pi/4$, AD (max), and (iii) estimated patterns from gSVD having the highest absolute correlation with the true patterns among the two pattern-pairs with angular distances nearest to 0, AD (0). Figure 7 (a)-(b) are the boxplots for the gene and protein patterns of the first pattern-pair respectively, while Figure 7 (c)-(d) are the boxplots for the gene and protein patterns of the second pattern-pair respectively. From the figures, the correlation was lower for the genes (maximum correlation 0.6) than the proteins (maximum correlation 0.8). There was no indication that the angular distance improved the strength of correlation between the estimated patterns from gSVD and the true patterns.

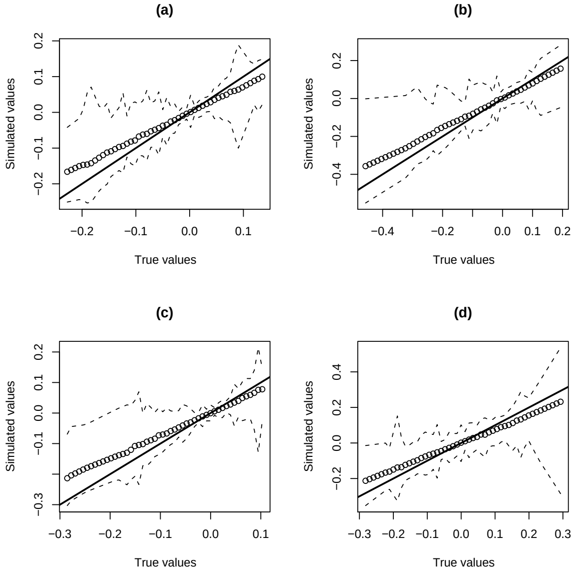

Figure 5: True pattern-pairs versus estimated pattern-pairs from CFA via SVD (250 simulations with $n = 59$ samples). (a) and (b) are the gene and protein patterns of the first pattern-pair respectively, while (c) and (d) are the gene and protein patterns of the second pattern-pair respectively. The solid line is the line-of-identity, the broken line is the interpolated 5-th and 95-th percentile of the estimated patterns from 250 simulations, while the circles are their interpolated means.

6 NCI data results of CFA (Molecular Function)

This section contains the results of CFA on NCI data. The number of GO terms and the corresponding number of enriched GO terms for the genes were 743 and 38 for the first gene patterns, 665 and 37 for the second gene patterns, and 784 and 16 for the third gene patterns. There were altogether 57 enriched GO terms and half of them (25 GO terms) were also interesting in another pattern-pair. Table 1 shows the top 10 most enriched GO terms from the gene patterns for each pattern-pair. There were altogether 23 unique GO terms.

Similar to CFA for biological process, we validated whether the pattern-pairs from CFA for molecular function gave coherent signal. Figure 8 shows GO terms associated with the top 10 proteins were lower than those from the bottom 10 and this difference was insignificant (p-value =0.454 using Wilcoxon test). This suggested that the pattern-pairs were discordant but not significant. When we considered the boxplot for each pattern-pair, we saw that the top 10 proteins had consistently higher median rank than the bottom 10, see Figure 9. This was not observed for gSVD.

GO ID	GO Term	Q-value(1)	Q-value(2)	Q-value(3)
GO:0004867	serine-type endopeptidase inhibitor activity	$4.19e-06$		
GO:0004866	endopeptidase inhibitor activity	$4.19e-06$		
GO:0030414	protease inhibitor activity	$4.19e-06$		
GO:0051015	actin filament binding	$1.49e-04$		
GO:0019838	growth factor binding	$6.38e-04$		
GO:0048154	S100 beta binding	$1.05e-03$		
GO:0005201	extracellular matrix structural constituent		$3.88e-06$	
GO:0005507	copper ion binding		$8.44e-06$	
GO:0030247	polysaccharide binding		$8.44e-06$	
GO:0005198	structural molecule activity		$1.17e-05$	
GO:0001871	pattern binding		$2.12e-05$	
GO:0005539	glycosaminoglycan binding		$2.63e-05$	
GO:0016717	oxidoreductase activity, acting on paired donors, with oxidation of a pair of donors resulting in the reduction of molecular oxygen to two molecules of water			0.000258
GO:0042802	identical protein binding			0.014500
GO:0019207	kinase regulator activity			0.014500
GO:0004295	trypsin activity			0.018200
GO:0030234	enzyme regulator activity			0.034300
GO:0017017	MAP kinase tyrosine/serine/threonine phosphatase activity			0.039700
GO:0005515	protein binding	$1.77e-04$	$3.20e-06$	
GO:0004857	enzyme inhibitor activity	$1.90e-06$		0.002900
GO:0005509	calcium ion binding		$1.94e-06$	0.017900
GO:0008092	cytoskeletal protein binding	$1.25e-06$	$1.94e-06$	0.000543
GO:0003779	actin binding	$1.25e-06$	$1.94e-06$	0.001130

Table 1: The first three pattern-pairs from CFA: The top 10 most enriched molecular function GO terms from genes.

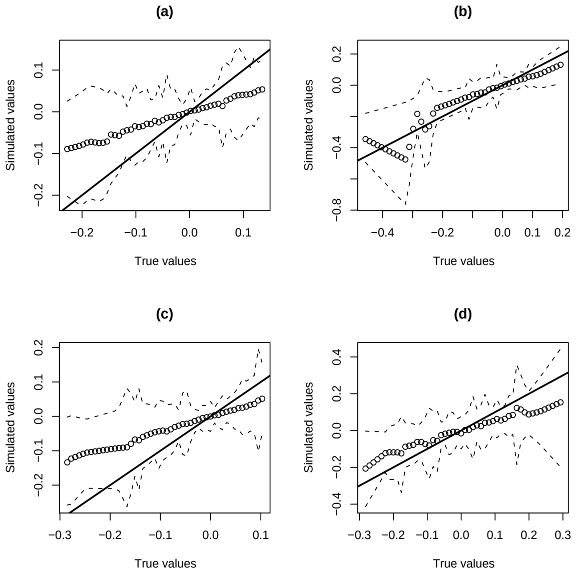

Figure 6: True pattern-pairs versus estimated patterns from gSVD having the highest absolute correlation with the true patterns (250 simulations with $n = 59$ samples). (a) and (b) are the gene and protein patterns of the first pattern-pair respectively, while (c) and (d) are the gene and protein patterns of the second pattern-pair respectively. The solid line is the line-of-identity, the broken line is the interpolated 5-th and 95-th percentile of the estimated patterns from 250 simulations, while the circles are their interpolated means.

7 NCI data results of gSVD (Biological Process)

This section contains the biological process results of gSVD on NCI data. The number of GO terms and the corresponding number of enriched GO terms for the genes were 1928 and 65 for the first gene patterns, 1854 and 3 for the second gene patterns, and 1991 and 71 for the third gene patterns. There were altogether 108 enriched GO terms and about 25% of them (28 GO terms) were also interesting in another pattern-pair. Table 2 shows the top 10 most enriched GO terms from the gene patterns. For the second pattern-pair, it had three enriched GO terms. There were altogether 14 unique GO terms.

We validated whether the pattern-pairs from gSVD for biological process gave coherent signal. Figure 10 shows GO terms associated with the top 10 proteins were lower than those from the bottom 10 and this difference was insignificant (p-value =0.130 using Wilcoxon test). This suggested that the pattern-pair information could be discordant but insignificant.

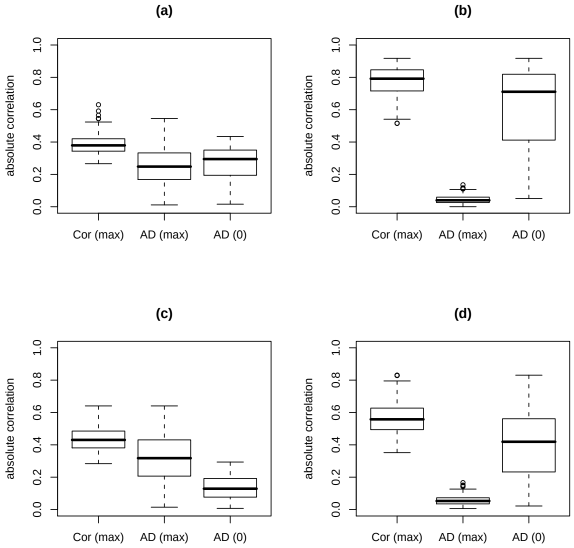

Figure 7: Boxplots of the correlation between the true patterns, and estimated patterns from gSVD. (a) and (b) are the gene and protein patterns of the first pattern-pair respectively, while (c) and (d) are the gene and protein patterns of the second pattern-pair respectively. Cor (max) corresponds to estimated patterns from gSVD having the largest absolute correlation with the true patterns. AD (max) corresponds to the estimated patterns from gSVD having the highest absolute correlation with true patterns among the two pattern-pairs with angular distances nearest to $\pi/4$, while AD (0) corresponds to the estimated patterns from gSVD having the highest absolute correlation with true patterns among the two pattern-pairs with angular distances nearest to 0.

GO ID	GO Term	Q-value(1)	Q-value(2)	Q-value(3)
GO:0001568	blood vessel development	$1.42e-05$		
GO:0007169	transmembrane receptor protein tyrosine kinase signaling pathway	$1.53e-05$		
GO:0048514	blood vessel morphogenesis	$1.53e-05$		
GO:0001944	vasculature development	$1.62e-05$		
GO:0048513	organ development			$1.00e-05$
GO:0009605	response to external stimulus			$1.02e-05$
GO:0009653	anatomical structure morphogenesis			$3.31e-04$
GO:0048731	system development	$1.91e-07$		$8.23e-08$
GO:0022610	biological adhesion			$5.35e-06$
GO:0032502	developmental process	$3.05e-09$	0.0257	$5.45e-07$
GO:0048856	anatomical structure development	$3.05e-09$		$1.35e-09$
GO:0007275	multicellular organismal development	$3.79e-07$	0.0436	$2.41e-08$
GO:0032501	multicellular organismal process	$1.03e-06$	0.0257	$5.48e-08$
GO:0007155	cell adhesion	$3.66e-05$		$5.35e-06$

Table 2: The first three pattern-pairs from gSVD with the smallest angular distances: The top 10 most enriched biological process GO terms from genes. For the second pattern-pair, it has three enriched GO terms.

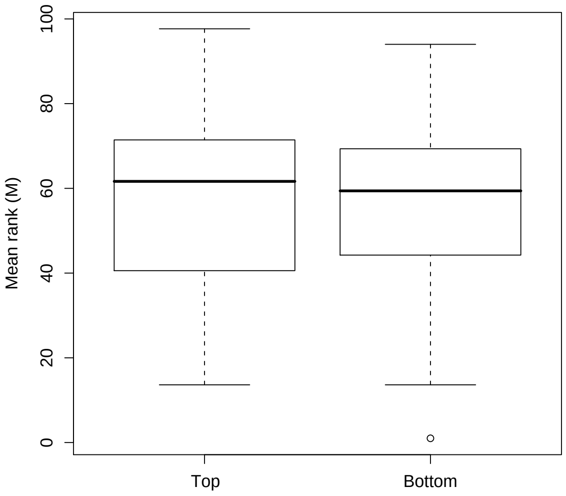

Figure 8: The boxplot of the average rank M of p-values from the GO analysis of the molecular function for genes that match with the protein's GO terms, which are from the top 10 and bottom 10 proteins. The GO analysis uses the first three pattern-pairs from CFA.

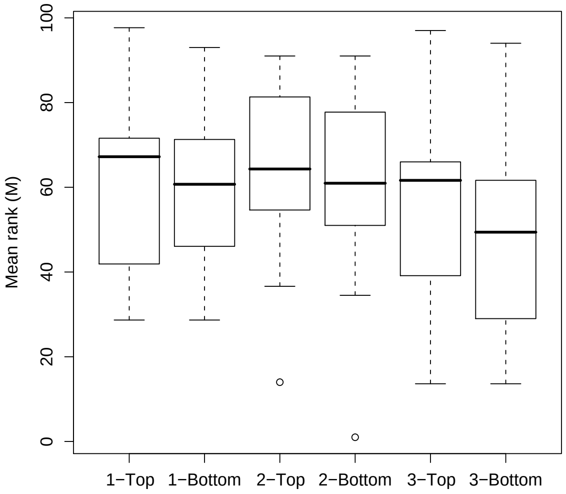

Figure 9: Similar to Figure 8 but we plot the average rank M of p-values for each pattern-pair.

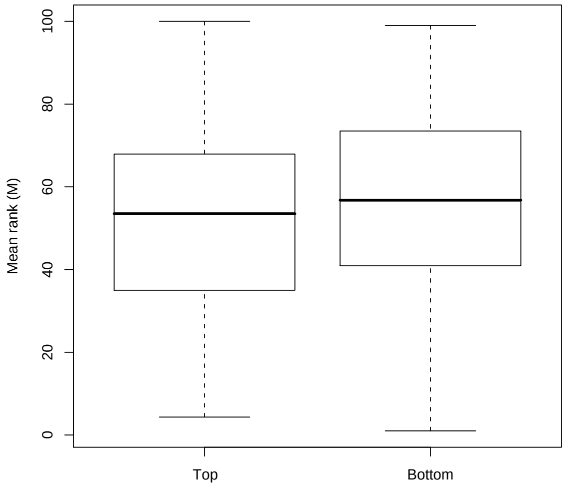

Figure 10: The boxplot of the average rank M of p-values from the GO analysis of the biological process for genes that match with the protein's GO terms, which are from the top 10 and bottom 10 proteins. The GO analysis uses the three pattern-pairs from gSVD with the three smallest angular distances.

8 NCI data results of gSVD (Molecular Function)

This section contains the molecular function results of gSVD on NCI data. The number of GO terms and the corresponding number of enriched GO terms for the genes were 833 and 17 for the first gene patterns, 768 and 1 for the second gene patterns, and 759 and 10 for the third gene patterns. There were altogether 21 enriched GO terms and about 30% of them (6 GO terms) were also interesting in another pattern-pair. Table 3 shows the top 10 most enriched GO terms from the gene patterns. For the second pattern-pair, it had one enriched GO terms. There were altogether 16 unqiue GO terms.

We validated whether the pattern-pairs from gSVD for molecular function gave coherent signal. Figure 11 shows GO terms associated with the bottom 10 proteins were similar with the top 10 (p-value =0.815 using Wilcoxon test). This suggested that the pattern-pair information could be discordant but insignificant.

GO ID	GO Term	Q-value(1)	Q-value(2)	Q-value(3)
GO:0008083	growth factor activity	0.0138		
GO:0008307	structural constituent of muscle	0.0273		
GO:0004033	aldo-keto reductase activity	0.0273		
GO:0047115	trans-1,2-dihydrobenzene-1,2-diol dehydrogenase activity	0.0273		
GO:0008201	heparin binding	0.0273		
GO:0005125	cytokine activity	0.0273		
GO:0005102	receptor binding			$1.56e-03$
GO:0016620	oxidoreductase activity, acting on the aldehyde or oxo group of donors, NAD or NADP as acceptor			$3.53e-03$
GO:0005520	insulin-like growth factor binding			$1.04e-02$
GO:0004030	aldehyde dehydrogenase [NAD(P)+] activity			$2.28e-02$
GO:0016903	oxidoreductase activity, acting on the aldehyde or oxo group of donors			$2.40e-02$
GO:0004857	enzyme inhibitor activity	0.0138		$1.46e-06$
GO:0004866	endopeptidase inhibitor activity	0.0273		$4.08e-07$
GO:0030414	protease inhibitor activity	0.0273		$4.08e-07$
GO:0004867	serine-type endopeptidase inhibitor activity	0.0273		$1.71e-05$
GO:0005201	extracellular matrix structural constituent		0.0138	$1.04e-02$

Table 3: The first three pattern-pairs from gSVD with the smallest angular distances: The top 10 most enriched molecular function GO terms from genes. For the second pattern-pair, it has one enriched GO terms.

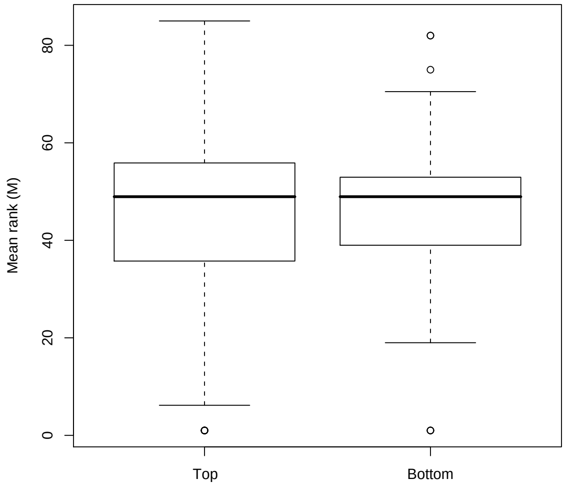

Figure 11: The boxplot of the average rank M of p-values from the GO analysis of the molecular function for genes that match with the protein's GO terms, which are from the top 10 and bottom 10 proteins. The GO analysis uses the three pattern-pairs from gSVD with the three smallest angular distances.

9 Compare CFA and gSVD on NCI data (Biological Process)

This section contains the results of comparing CFA and gSVD on NCI data, where the three pattern-pairs from gSVD had the highest correlation with the first three pattern-pairs from CFA. The number of GO terms and the corresponding number of enriched GO terms for the genes were 1975 and 96 for the first gene patterns, 1854 and 46 for the second gene patterns, and 2091 and 110 for the third gene patterns. There were altogether 172 enriched GO terms and about 30% of them (54 GO terms) were also interesting in another pattern-pair. Table 4 shows the top 10 most enriched GO terms from gene patterns. There were altogether 19 unqiue GO terms.

GO ID	GO Term	Q-value(1)	Q-value(2)	Q-value(3)
GO:0002504	antigen processing and presentation of peptide or polysaccharide antigen via MHC class II	$2.58e-05$		
GO:0050896	response to stimulus	$2.89e-05$		
GO:0030154	cell differentiation	$2.49e-04$		
GO:0048869	cellular developmental process	$2.49e-04$		
GO:0022610	biological adhesion	$3.99e-04$		
GO:0001568	blood vessel development		$4.36e-07$	
GO:0001944	vasculature development		$6.99e-07$	
GO:0048514	blood vessel morphogenesis		$1.69e-06$	
GO:0009888	tissue development			$4.03e-06$
GO:0051046	regulation of secretion			$9.72e-05$
GO:0042060	wound healing			$2.68e-04$
GO:0065008	regulation of biological quality			$2.68e-04$
GO:0007155	cell adhesion	$3.99e-04$	$1.69e-06$	
GO:0048513	organ development		$1.08e-07$	$2.68e-04$
GO:0007275	multicellular organismal development		$1.57e-07$	$1.14e-04$
GO:0032501	multicellular organismal process	$7.37e-06$	$7.06e-10$	$2.94e-07$
GO:0032502	developmental process	$1.68e-05$	$1.64e-09$	$9.72e-05$
GO:0048856	anatomical structure development	$2.89e-05$	$1.64e-09$	$1.65e-05$
GO:0048731	system development	$2.49e-04$	$2.52e-08$	$1.09e-04$

Table 4: The three pattern-pairs from gSVD having the highest absolute correlation with the first three pattern-pairs from CFA: The top 10 most enriched biological process GO terms from genes.

We validated whether the pattern-pairs from gSVD for biological process gave coherent signal. Figure 12 shows GO terms associated with the top 10 proteins were higher than those from the bottom 10 and this difference was significant (p-value =0.019 using Wilcoxon test).

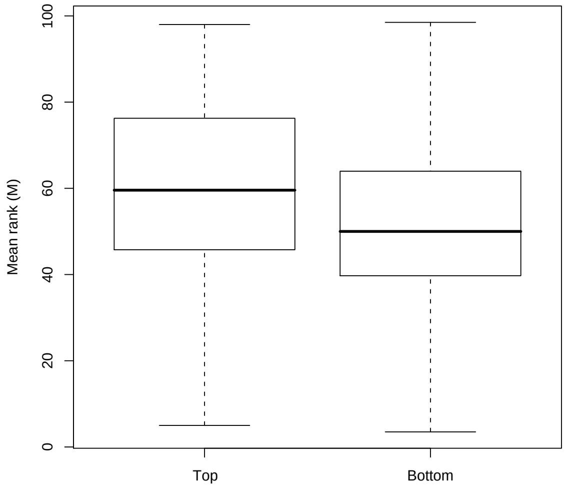

Figure 12: The boxplot of the average rank M of p-values from the GO analysis of the biological process for genes that match with the protein's GO terms, which are from the top 10 and bottom 10 proteins. The GO analysis uses the three pattern-pairs from gSVD which have the highest absolute correlation with the first three pattern-pairs from CFA.

10 Compare CFA and gSVD on NCI data (Molecular Function)

The number of GO terms and the corresponding number of enriched GO terms for the genes were 732 and 3 for the first gene patterns, 803 and 15 for the second gene patterns, and 799 and 9 for the third gene patterns. There were altogether 21 enriched GO terms and about 10% of them (2 GO terms) were also interesting in another pattern-pair. Table 5 shows the top 10 most enriched GO terms from gene patterns. For the first and third pattern-pairs, they had three and nine enriched GO terms respectively.There were altogether 21 unqiue GO terms.

We validated whether the pattern-pairs from gSVD for molecular function gave coherent signal. Figure 13 shows GO terms associated with the top 10 proteins were similar with the bottom 10 (p-value =0.891 using Wilcoxon test).

Figure 14 suggests that only CFA had consistent concordance between the gene and protein GO terms in all three pattern-pairs.

GO ID	GO Term	Q-value(1)	Q-value(2)	Q-value(3)
GO:0032395	MHC class II receptor activity	$4.94e-05$		
GO:0043548	phosphoinositide 3-kinase binding	$8.23e-03$		
GO:0005520	insulin-like growth factor binding	$2.86e-02$		
GO:0004857	enzyme inhibitor activity		0.000831	
GO:0004860	protein kinase inhibitor activity		0.015500	
GO:0005507	copper ion binding		0.015500	
GO:0019210	kinase inhibitor activity		0.015500	
GO:0003779	actin binding		0.015500	
GO:0019207	kinase regulator activity		0.015500	
GO:0046870	cadmium ion binding		0.015500	
GO:0005515	protein binding		0.015500	
GO:0008201	heparin binding		0.015500	
GO:0005509	calcium ion binding			0.00328
GO:0004653	polypeptide N-acetylgalactosaminyltransferase activity			0.03490
GO:0030296	protein tyrosine kinase activator activity			0.03490
GO:0005201	extracellular matrix structural constituent			0.03680
GO:0005200	structural constituent of cytoskeleton			0.03710
GO:0008376	acetylgalactosaminyltransferase activity			0.03990
GO:0008083	growth factor activity			0.03490
GO:0005198	structural molecule activity			0.03710
GO:0005102	receptor binding		0.001050	0.03490

Table 5: The three pattern-pairs from gSVD having the highest absolute correlation with the first three pattern-pairs from CFA: The top 10 most enriched molecular function GO terms from genes. For the first and third pattern-pairs, they have three and nine enriched GO terms respectively.

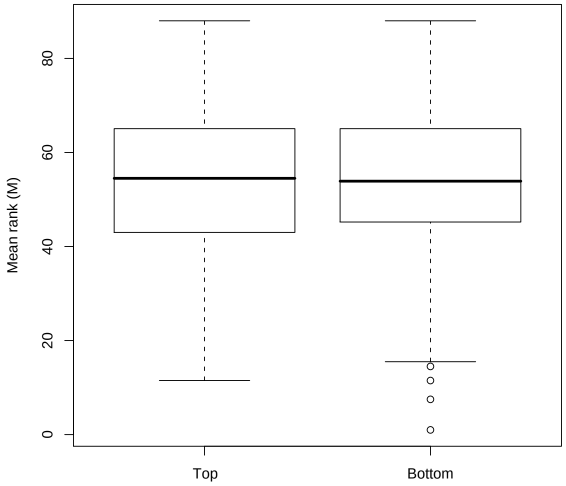

Figure 13: The boxplot of the average rank M of p-values from the GO analysis of the molecular function for genes that match with the protein's GO terms, which are from the top 10 and bottom 10 proteins. The GO analysis uses the three pattern-pairs from gSVD which have the highest correlation with the first three pattern-pairs from CFA.

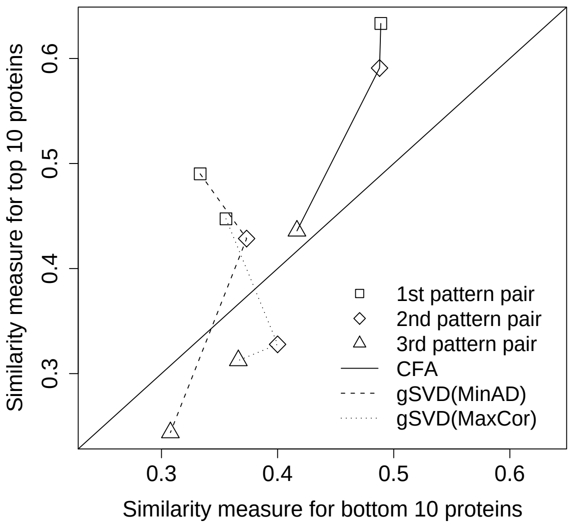

Figure 14: The proportion of nodes from the gene's induced graph overlapping with the nodes from the protein's induced graph (similarity measure) for the top and bottom 10 proteins of CFA and gSVD. The diagonal line is where the values from the x-axis and y-axis are equal. gSVD(MinAD): gSVD with the smallest angular distances, gSVD(MaxCor): gSVD having the highest correlation with the first three pattern-pairs from CFA. These are results based on the GO analysis of the molecular function.
